# Dietary supplementation and the quality of life of retired football players

**DOI:** 10.1186/1550-2783-9-S1-P28

**Published:** 2012-11-19

**Authors:** Robert A Sinnott, Rolando L Maddela, Sejong Bae, Talitha Best

**Affiliations:** 1 Research and Development, Mannatech, Incorporated, 600 S Royal Lane Suite 200, Coppell, TX 75019, USA; 2 Department of Biostatistics, UNTHSC- School of Public Health, 3500 Camp Bowie Blvd. Fort Worth, TX 76107, USA; 3 Nutritional Physiology Research Centre, Sansom Institute for Health Research, University of South Australia, Adelaide, South Australia

## Background

Studies suggest that playing professional football can impact the health of the athlete and concerns are raised that they may experience negative health consequences that may affect their quality of life when they retire. The purpose of this exploratory investigation is to determine the effects of dietary supplementation on the quality of life of retired football players.

## Methods

Questionnaires were completed by 15 ambulatory retired football players with the average age of 49.6 (±8.2) years and average professional football career of 7.6 (±3.2) years. In this open label study, the subjects had daily intake of the following supplements for 6 months: Fish oil with vitamin D3, antioxidant, natural vitamin and mineral supplement, glyconutrient and a phytosterol-amino acid complex. Outcome measures included “Healthy Days Measures” (CDC HRQOL-4), WHO Quality of Life (WHOQOL-BREF), Profile of Mood States (POMS) and Memory Functioning Questionnaire (MFQ). Self-assessments of pain of joints and extremities as well as range of motion were also collected using a questionnaire. Mean differences were assessed between baseline and each data collection point at 1, 3 and 6 months.

## Results

Statistically significant differences from baseline were obtained in key outcome measures. CDC HRQOL general health rating showed improvement at month 1 (p=0.008) and sustained to month 6 (p<0.0001). There was increased number of healthy days per month related to physical health and mental health and the improvement in the number of mental health days was significant at 6 months (p=0.029). WHOQOL-BREF showed improvement on the rating of quality of life at 6 months (p=0.038) and satisfaction with health in all measurement points (p<0.05). Both the Physical and Psychological Domains showed significant improvement at 6 months (p<0.05). General Rating of Memory using the MFQ showed significant improvement at 3 and 6 months (p<0.05). The POMS showed that the participants rated the Vigor scale significantly higher at 3 months compared to the baseline (p=0.024). Self-assessment of pain showed decreasing trends but only the elbow and knee pain showed statistically significant improvement at 1 and 3 months, (p<0.05). There were no adverse events related to the supplementation.

## Conclusions

This preliminary study demonstrates that multiple dietary supplementations enhanced the quality of life of this special group of retired football players. However, a larger well controlled clinical trial is needed to determine whether these findings can be replicated not only in this special population but also in other group of retired athletes.

## Competing interests

R. A. Sinnott and R. L. Maddela are employees of Mannatech, Incorporated. The other authors do not have any conflict of interest.

